# Improving the sensitivity of long read overlap detection using grouped short *k*-mer matches

**DOI:** 10.1186/s12864-019-5475-x

**Published:** 2019-04-04

**Authors:** Nan Du, Jiao Chen, Yanni Sun

**Affiliations:** 1 Department of Computer Science and Engineering, Michigan State University, East Lansing, 48824 MI USA; 2 Electronic Engineering Department, City University of Hong Kong, Hong Kong SAR, China

**Keywords:** Group hit criteria, Third-generation sequencing, Overlap detection, Metagenomics

## Abstract

**Background:**

Single-molecule, real-time sequencing (SMRT) developed by Pacific BioSciences produces longer reads than second-generation sequencing technologies such as Illumina. The increased read length enables PacBio sequencing to close gaps in genome assembly, reveal structural variations, and characterize the intra-species variations. It also holds the promise to decipher the community structure in complex microbial communities because long reads help metagenomic assembly. One key step in genome assembly using long reads is to quickly identify reads forming overlaps. Because PacBio data has higher sequencing error rate and lower coverage than popular short read sequencing technologies (such as Illumina), efficient detection of true overlaps requires specially designed algorithms. In particular, there is still a need to improve the sensitivity of detecting small overlaps or overlaps with high error rates in both reads. Addressing this need will enable better assembly for metagenomic data produced by third-generation sequencing technologies.

**Results:**

In this work, we designed and implemented an overlap detection program named GroupK, for third-generation sequencing reads based on grouped *k*-mer hits. While using *k*-mer hits for detecting reads’ overlaps has been adopted by several existing programs, our method uses a group of short *k*-mer hits satisfying statistically derived distance constraints to increase the sensitivity of small overlap detection. Grouped *k*-mer hit was originally designed for homology search. We are the first to apply group hit for long read overlap detection. The experimental results of applying our pipeline to both simulated and real third-generation sequencing data showed that GroupK enables more sensitive overlap detection, especially for datasets of low sequencing coverage.

**Conclusions:**

GroupK is best used for detecting small overlaps for third-generation sequencing data. It provides a useful supplementary tool to existing ones for more sensitive and accurate overlap detection. The source code is freely available at https://github.com/Strideradu/GroupK.

## Background

The increased read length enables third-generation sequencing to close gaps in genome assembly [1, 2], reveal structural variations [3], and quantify gene isoforms with higher accuracy [4] in transcriptomic sequencing. In addition, using long reads holds promise in revealing the microbial community structure and deciphering intra-species variation for microbial communities [5, 6].

Genome assembly using third-generation sequencing data requires dedicated methods and tools. Existing genome assembly tools mainly utilize two types of graph models: overlap graph and de Bruijn graph. When the error rate is low, de Bruijn graph has the theoretical advantage that the graph size does not increase significantly with the sequencing coverage, which is usually high for Illumina datasets. For third-generation sequencing data, the high error rate and low coverage make the overlap graph a sensible choice for genome assembly [7]. A key step in constructing the overlap graph is to identify read pairs that share overlaps, which indicates that these reads are sequenced from the same loci in the underlying genome. Although there are a number of sequence alignment programs available for conducting overlap alignment [8, 9], a majority of them rely on dynamic programming and are too computationally expensive for high throughput sequencing data. Due to high error rates, existing short read overlap detection software using BWT (Burrows-Wheeler transform) or hash table [10, 11] cannot be directly applied to long reads.

### Related work

Two strategies are currently being employed to detect overlaps for error-prone long reads. One strategy tries to correct sequencing errors in PacBio (Pacific Biosciences) and ONT (Oxford Nanopore) data before overlap detection. There exist a number of sequencing error correction tools [7, 12]. Some of them rely on hybrid sequencing, which requires preparation of at least two sequencing libraries and several types of sequencing runs and thus is not cost-effective for many applications. Others conduct error correction using long reads only. One representative method is described in Chin et al.’s hierarchical genome-assembly process HGAP [12], whose performance improves with higher read coverage. It is worth noting that for alignment-based error correction methods such as the one in HGAP, an important step is to identify reads that can be aligned quickly. Essentially, techniques used for overlap detection can be used for alignment detection as well.

The second category bypasses the difficulty of error correction and identifies overlaps using raw reads. Various approximate similarity search methods have been applied on PacBio and ONT data [13]. They generally follow seed-chain-align procedure [14]. Seed-based filtration step plays an essential role in controlling the trade-off between sensitivity and computational efficiency. Usually, these methods use short string matches as the filtration step. A short string or *k*-mer match requires exact matches of *k* consecutive characters between two sequences. Intuitively, overlapping reads tend to share more common *k*-mers than non-overlapping reads. Strategies that can quickly find the number of shared *k*-mers can thus be applied. In this section, we summarize the main strategies of several state-of-the-art overlap detection tools. We highlight the differences between our method and the existing ones in the following section.

MHAP [15], Minimap [14, 16], and DALIGNER [17] all use *k*-mer matches for identifying candidate overlapping pairs. Due to the high error rate, usually only short *k*-mers will be applied in order to achieve high sensitivity. However, identifying short *k*-mer matches between all pairs of reads is computationally expensive. Thus, the leading tools employed different data structures and algorithms for estimating *k*-mer-based similarity. MHAP converts long reads into sets of *k*-mers and sketches using minHash. Then the similarity between reads is estimated using the compact sketches. Minimap also uses a compact representation of the original reads by keeping minimizers rather than all possible *k*-mers of a read. Then collinear *k*-mers will be clustered and used for checking possible overlaps. DALIGNER directly sorts *k*-mers based on their positions and then utilizes merge sort to identify the number of shared *k*-mers. As the sorting is cache efficient, DALIGNER is practically very efficient.

BLASR [18] was initially designed for mapping PacBio reads to a reference genome. It is also widely used as an overlap detection tool for PacBio data. BLASR uses BWT and FM index to identify short *k*-mer matches and then clusters *k*-mer matches within a given distance range. The clustered *k*-mers are ranked based on a function of the *k*-mer frequency. Only highly ranked clusters will be kept for downstream analysis.

Being different from the above tools, GraphMap [19] uses spaced seeds that allow matches of non-consecutive characters. Spaced seeds were initially used in homology search for improving the trade-off between sensitivity and filtration efficiency [20–22]. In particular, spaced seeds containing the pattern “11*” have high sensitivity in capturing homologous protein-coding genes because of the codon structure. However, designing optimal spaced seeds (i.e., deciding the positions of the wildcard characters) is NP-hard [23, 24]. GraphMap empirically chooses two spaced seeds. Ideally, different sets of seeds may be designed for input data of different error profiles.

### Overview of our work

The high error rates and also the different error profiles of PacBio and ONT data motivate us to use a more flexible seeding strategy called group hit criteria [25], which define a group of possibly overlapping *k*-mers satisfying statistically derived distance constrains. For brevity, we will call the *k*-mers set satisfying the group hit criteria as a “group seed”. A group seed was initially proposed and used for homology search. Given the error profiles, such as the estimated indels and mismatch probabilities, thresholds for grouping short *k*-mers can be computed using the waiting time distribution and the one-dimensional random walk [25]. A group seed can effectively handle all types of errors and is ideal to detect small overlaps. With group seeds, we can achieve high sensitivity using short *k*-mers (e.g., 9-mer) while still maintaining a desirable specificity.

In this work, we employ group seeds for detecting overlapping long reads for genome assembly. Our implementation, named GroupK, provides a complementary tool to existing methods for detecting small overlaps or overlaps compounded by high error rates of both reads. This ability enables our tool a sensible choice for genome assembly in metagenomic data sequenced by third-generation sequencing platforms. As these community samples usually contain microorganisms with heterogeneous coverage, being able to identify small overlaps will be very important for reconstructing genomes of rare species.

## Methods

**Fig. 1:**
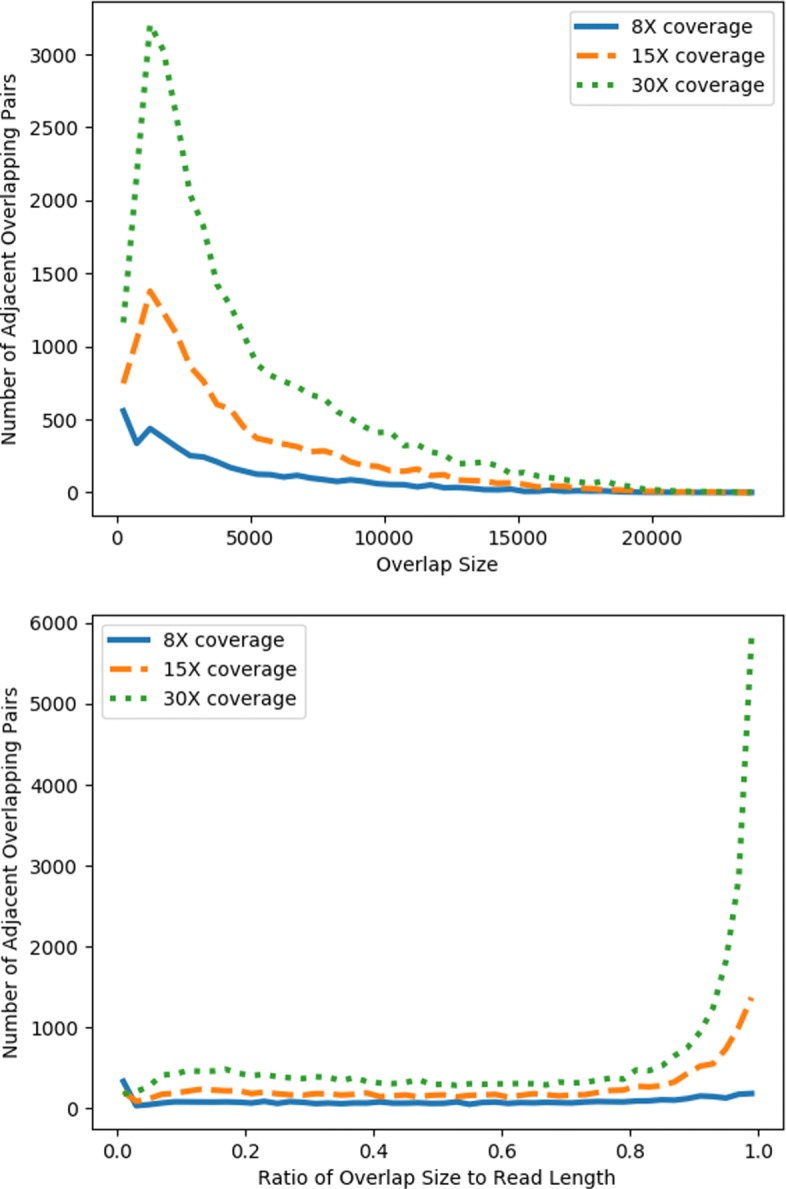
Histograms of irreducible overlap sizes (top) and the ratio of overlap size to the read length (bottom) when comparing adjacent overlapping reads on simulated PacBio *E. coli* datasets given different coverages. The bin width for the overlap size is 500. For the 30X dataset, the average read length is 8366 and the number of reads is 16644. For the 15X dataset, the average read length is 8253 and the number of reads is 8436. For the 8X dataset, the average read length is 8414 and the number of reads is 4413

GroupK is designed for improving the sensitivity of detecting small overlaps or overlaps with low identity. Currently, third-generation sequencing data still has high error rates. The overlapping regions formed by two error-prone long reads can have lower sequence identity than mapping a long read against a reference genome. Figure 1 presents the histogram of the overlap size and the corresponding ratio of overlap size to the read length between two adjacent reads. The reads are simulated using PBSIM [26] from *E. coli* with three different coverages. As we know the position of each simulated read in the genome, the overlap size can be easily decided. Note that a read can form overlaps with multiple reads sequenced from the same region. However, the two figures are generated using overlaps between two adjacent reads, which define an “irreducible” edge [27] in an overlap graph. Thus, these overlaps can decide the continuity of the final genome assembly. The figures show that there are still substantial regions with small overlaps. For example, there are 45.46%, 34.76%, and 31.19% of the overlaps shorter than the 50% of the read length for data with coverage of 8X, 15X, and 30X, respectively. It will be ideal to detect relatively small overlaps to fully take advantage of the long reads for generating more complete assemblies.

### Pipeline

**Fig. 2:**
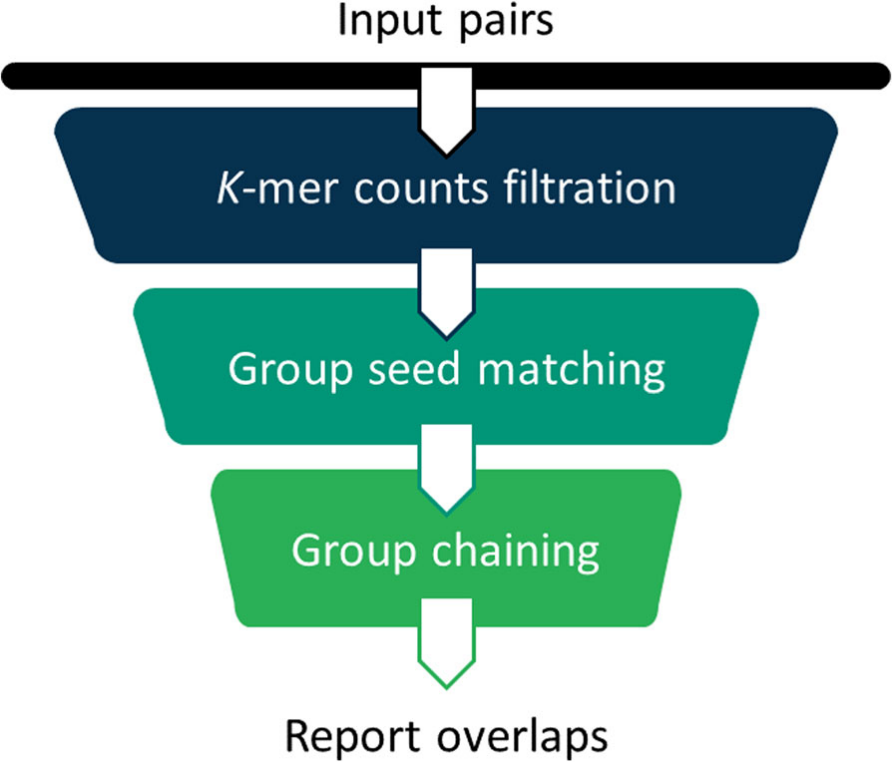
The pipeline of GroupK

**Fig. 3:**
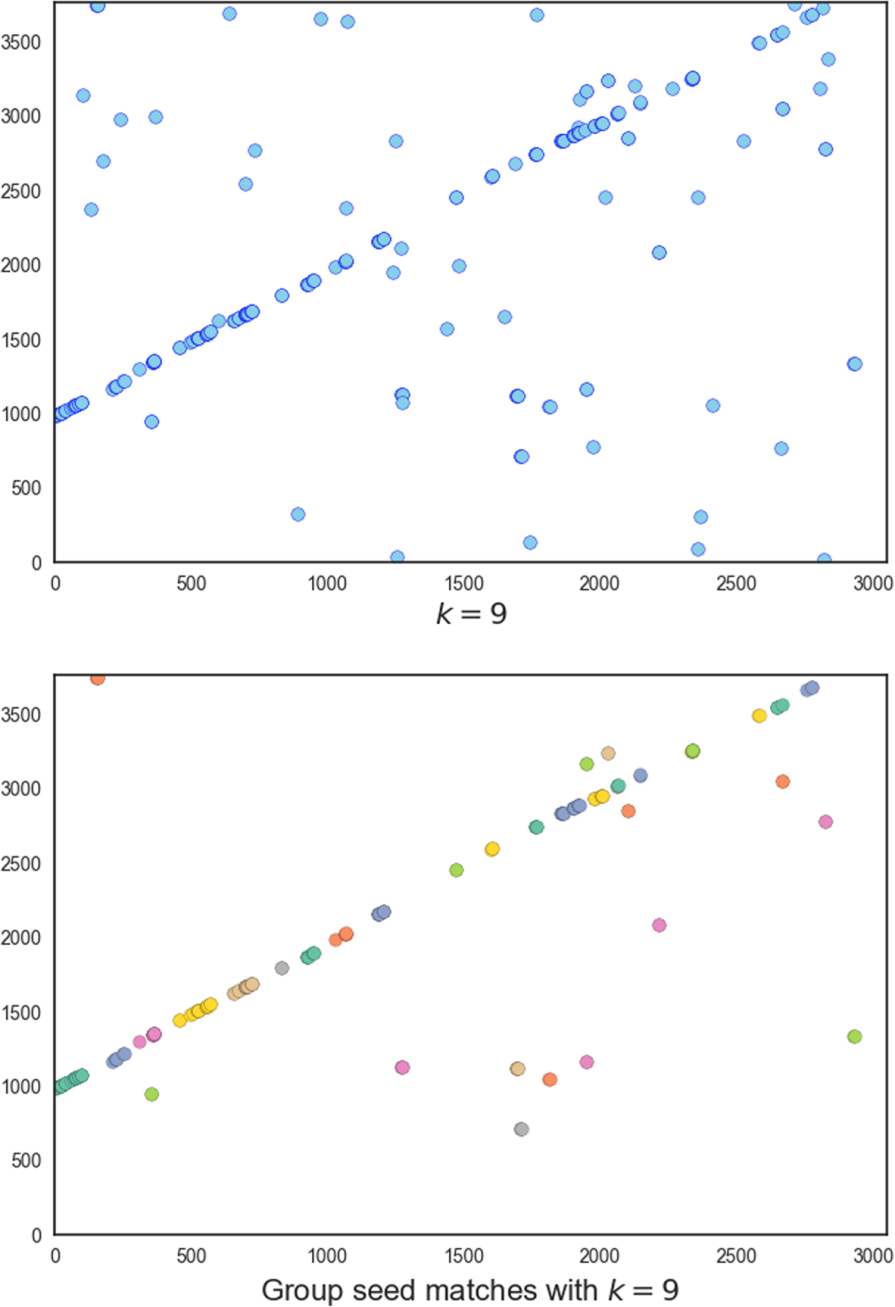
The dot plot of *k*-mer hits and group seeds matches for one overlapping pair from the *E. coli* PacBio dataset used in Results. Each dot is a *k*-mer hit. The x-axis and y-axis show the locations of the hits on the reads. The overlap region is roughly from 0 to 2800 on the x-axis, and from 1000 to 3800 on the y-axis. Top: all 9-mer hits. Bottom: 9-mer hits that passed the group hit criteria. A group seed is represented by closely located dots of the same color

Identifying small overlaps is computationally difficult. Thus, we use a carefully designed hierarchical filtration strategy to distinguish true overlapping reads from non-overlapping ones. The pipeline of GroupK consists of three key steps: filtration, group seed matching, and chaining (Fig. 2). Filtration is used to reduce the search space by quickly identifying read pairs sharing a minimum number of *k*-mers. High insertion/deletion error rates tend to produce short *k*-mer matches on different diagonals. Thus, we adopt group seed matching to identify a group of short *k*-mer matches in close proximity. There are two types of distance constraints. 1) The distance (number of nucleotides) between the *k*-mers on x-axis and y-axis must be smaller than a given threshold; 2) the diagonal distance, which is the difference of the diagonals of two *k*-mer matches, must be within a given range. Chaining is used to estimate the final overlap region. Figure 3 shows that applying group seed can remove a large number of random *k*-mer hits while keeping the *k*-mer matches within the overlapping region. When *k*=15, there are only two hits, and it is difficult to determine whether there is an overlap. When *k*=9, there is clearly a chain formed by hits in the overlapping region. However, there are also a large number of random hits. With group seed matching criteria, most of the random 9-mer hits are filtered out. So the downstream analysis becomes more straightforward.

### Estimate the expected number of *k*-mers for the filtration stage

In this section, we analyze the expected number of random *k*-mer hits between two reads and also *k*-mer hits in overlaps. The analysis will be used for determining the *k*-mer size and also other parameters for the filtration stage. Given two reads (*S*_1_ and *S*_2_) with length *L* and error rate *ε*, we want to determine how many *k*-mer hits we expect to find between *S*_1_ and *S*_2_.

We first consider the case that *S*_1_ and *S*_2_ are not related (no overlap). By assuming that the bases in *S*_1_ and *S*_2_ are randomly distributed, the expected number of random *k*-mer matches *E*[*X*_*r*_] is roughly:

1 $$ E\left[X_{r}\right] = \left(\frac{1}{|\Sigma|}\right)^{k} \cdot L^{2}  $$

Note that this equation is different from the expected number of shared *k*-mers in MHAP [15] because we distinguish *k*-mer hits based on their locations rather than the *k*-mers themselves. Also, we assume that overlapping *k*-mers are independent.

**Fig. 4:**
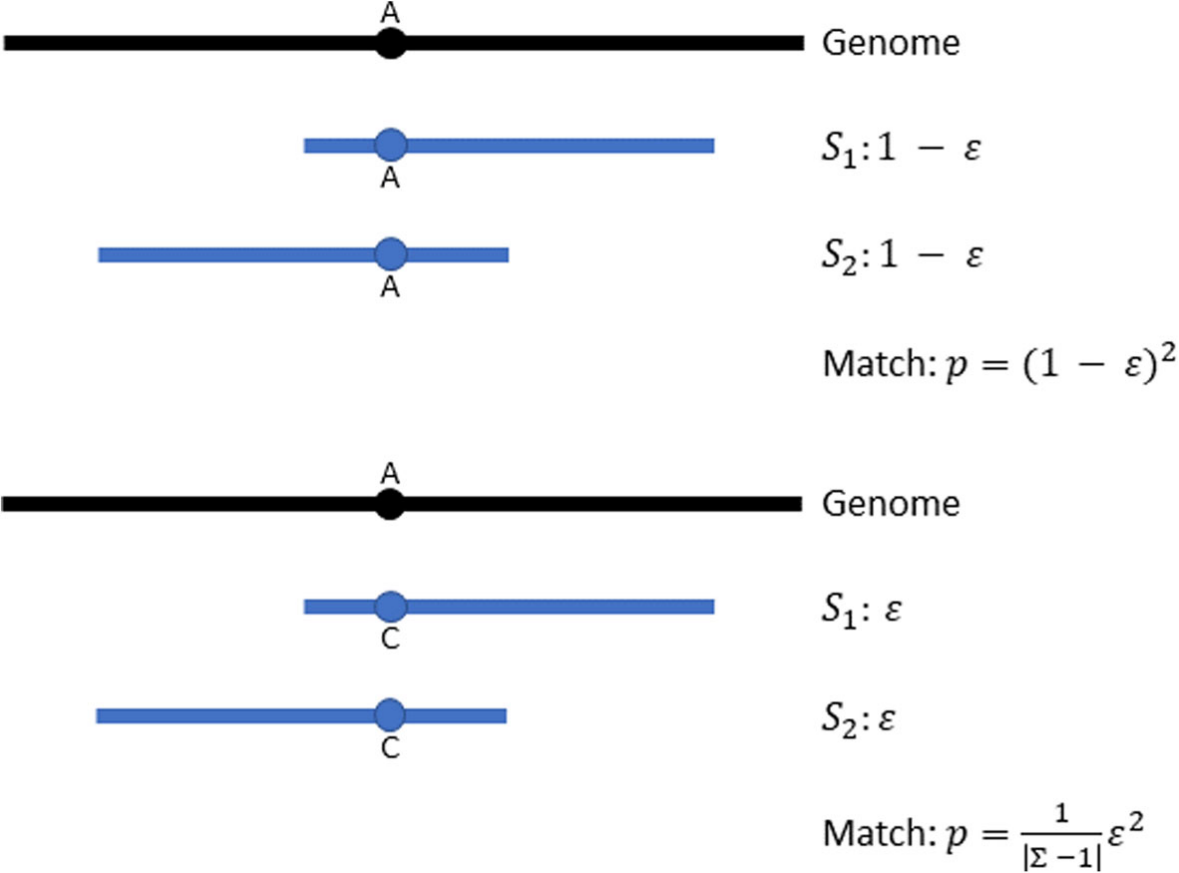
Two cases contributed to the identical characters at an aligned position in the overlapping region of *S*_1_ and *S*_2_. *S*_1_ and *S*_2_ are two reads sequenced from the same region of the underlying genome and form an overlap. Top: both bases on *S*_1_ and *S*_2_ are correct, forming a match. Bottom: both bases on *S*_1_ and *S*_2_ are sequencing errors, and substituted by the same character

In the second case of *S*_1_ and *S*_2_ forming an overlap, we estimate the expected number of *k*-mer matches in the overlap by first computing *P*_o_, which is the probability of observing a *k*-mer match within the overlap. In the case of no sequencing error (i.e. *ε*=0), the probability of observing a *k*-mer match at an aligned position in an overlap is simply 1.0. But in the practical case of *ε*>0, we need to consider two scenarios in order to determine the probability of observing two identical characters at an aligned position in the overlap: 1) the characters from *S*_1_ and *S*_2_ are correct; 2) the two characters from *S*_1_ and *S*_2_ are errors and are randomly substituted by the same character. The two cases are visualized in Fig. 4. So the probability is given by [15]:

2 $$ P_{\mathrm{o}} = \left[(1-\varepsilon)^{2} + \varepsilon^{2}\frac{1}{|\Sigma| - 1}\right]^{k}  $$

Considering both random *k*-mer matches and *k*-mer matches in an overlap, the expected number of shared *k*-mers between two overlapping reads is estimated by:

3 $$ E\left[X_{o}\right] = P_{\mathrm{o}} \cdot M + \left(\frac{1}{|\Sigma|}\right)^{k} \cdot L^{2}  $$

*M* is the size of the overlap. Note that the above equation slightly over-counts the number of *k*-mer hits in an overlap because the random *k*-mer hits inside the overlap may be counted twice with probability $P_{\mathrm {o}} \cdot \left (\frac {1}{|\Sigma |}\right)^{k}$. (Also, we assume that the probabilities of substitution and insertion/deletion are on the same order and thus do not distinguish them in the above equation.)

**Fig. 5:**
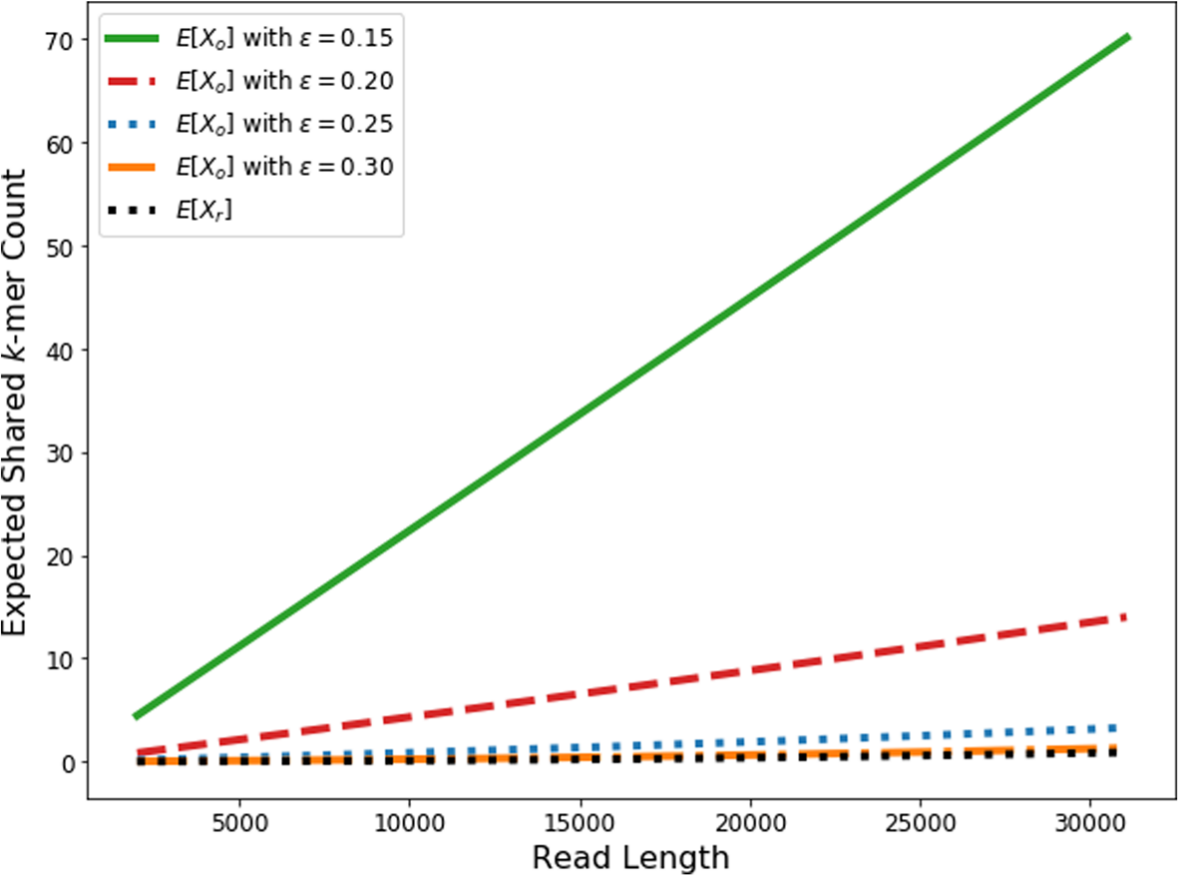
The change of *E*[*X*_*o*_] and *E*[*X*_*r*_] (y-axis) with the increase of the read length (x-axis), which is obtained from a real PacBio dataset. The overlap size is set as the 1/4 of the read length as we focus on identifying the hard case of small overlaps. *k*=15

As we are mainly interested in finding small overlaps or overlaps with low sequence identity, we plot the expected number of *k*-mer hits with the overlap size being 1/4 of the read size in Fig. 5. In order to plot the figure, we compute *E*[*X*_*o*_] and *E*[*X*_*r*_] using the read lengths from a 15X *E. coli* PacBio dataset (the data of our second experiment in Results). We only consider reads of length above 2,000. These figures allow us to choose the appropriate threshold for *k*-mer-counting based filtration. For example, Fig. 5 shows the expected number of 15-mers between reads of different error rates. *E*[*X*_*r*_] started as 4 when *ε*=0.15. And, for larger *ε*, *E*[*X*_*r*_] is even smaller. Thus, our default filtration threshold is two 15-mers in order to ensure high filtration sensitivity. The implementation details of the *k*-mer counting stage can be found towards the end of the “Methods” section.

### Group hit criteria

Sequencing errors tend to produce short *k*-mer matches. In addition, the insertion/deletion errors lead to *k*-mer matches on different diagonals. Thus, instead of using relatively long *k*-mers (such as 15 or 16-mers) as existing tools do, we use a group of short *k*-mers (such as 9-mer) to accommodate the high insertion or deletion error rates. A group seed is a set of possibly overlapping *k*-mer hits with statistically calculated constraints. The region containing group seeds is more likely to be inside an overlap than a single *k*-mer hit. Reference [25] first introduced the group hit criteria and also derived the method to calculate the criteria statistically. We apply their method for overlap detection.

Assume that we have two reads *S*_1_ and *S*_2_ of length *m* and *n*, respectively. The numbers of *k*-mers at different positions in *S*_1_ and *S*_2_ are *m*−*k*+1 and *n*−*k*+1, respectively. A *k*-mer hit at position (*i*,*j*) is defined by *S*_1_[*i*…*i*+*k*−1]=*S*_2_[*j*…*j*+*k*−1], where *i*≤*m*−*k*+1 and *j*≤*n*−*k*+1. For two *k*-mer hits at (*i*_1_,*j*_1_) and (*i*_2_,*j*_2_), their inter-seed distance *D*((*i*_1_,*j*_1_),(*i*_2_,*j*_2_)) is the maximum of |*i*_2_−*i*_1_| and |*j*_2_−*j*_1_|. The *k*-mer diagonal of a *k*-mer hit at (*i*,*j*), *d*(*i*,*j*), is defined as *j*−*i*.

With these notations, the goal is to solve the following inequalities given confidence level 1−*α* defined by significance level *α*:

4 $$\begin{array}{*{20}l} D\left(\left(i_{1}, j_{1}\right), \left(i_{2}, j_{2}\right)\right) &\leq \rho \end{array} $$

5 $$\begin{array}{*{20}l} |d\left(i_{1}, j_{1}\right) - d\left(i_{2}, j_{2}\right)| &\leq \delta  \end{array} $$

*ρ* and *δ* are integers we need to define the group hit criteria. For example, when *α*=0.05, our goal is to derive *ρ* and *δ* so that with 95% chance the inter-seed distance and the diagonal shift between two *k*-mers in overlapping reads are at most *ρ* and *δ*, respectively.

**Constraint on *k*-mer distance** We followed the model described in the article [25]. Runs of head in *n*-independent Bernoulli trials are used to model *k*-mer matches, with the probability *p* for a match and (1−*p*) for a mismatch. In this model, a *k*-mer match can be treated as *k* consecutive match runs with probability of *p*^*k*^. From the waiting time distribution [28, 29], the probabilities of the inter-seed distance *x* of two *k*-mers in an overlap are:

6 $$ {\begin{aligned} \mathcal{P}\left[D_{k} = x\right] =\left\{ \begin{array}{lr} 0 &\text{for}\ 0\leq x < k\\ p^{k} &\text{for}\ x = k \\ (1-p)p^{k}\left(1 - \sum_{i = 0}^{x -k - 1} \mathcal{P}\left[D_{k} = i\right]\right) &\text{for}\ x > k \end{array} \right. \end{aligned}}  $$

With the confidence level 1−*α*, *ρ* can be solved using following equation:

7 $$ \mathcal{P}\left[D_{k} \leq \rho\right] = 1- \alpha  $$

In the actual implementation we use *α*=0.05. Thus, there is 95*%* chance that the inter-seed distance of the two seeds in an overlap is less than the *ρ* calculated using Eq. 7.

**Constraint on *k*-mer diagonal distance** The diagonal shift between two *k*-mers, |*d*(*i*_1_,*j*_1_)−*d*(*i*_2_,*j*_2_)|, in two overlapping reads is caused by insertions and deletions. Note that the insertions and deletions are defined by comparing two reads, not between a read to a reference genome. So the insertion rate and deletion rate are treated equally.

The diagonal shift between two *k*-mer hits can be modeled by a discrete one-dimensional random walk model [25, 30]. The diagonal shift starts from 0. Let the steps of the random walk be *l*. Assume that the insertion and deletion rate is *q* across the whole read. Thus, the probability of a diagonal change is *q*, and the probability of staying in place is 1−2*q*. Also, we assume that in *l* steps, there are *n*_*i*_ inserted nucleotides (increase shift), *n*_*d*_ deleted nucleotides (decrease shift), and *n*_*m*_ matched nucleotides (no impact on shift). If the final diagonal shift is *i*, we have the following equations:

8 $$ \left\{\begin{array}{l} n_{i} + n_{d} + n_{m} = l\\ n_{i} - n_{d} = i \end{array}\right.  $$

Reference [25] calculated the probability of obtaining a diagonal shift *i* after *l* steps in the random walk. According to Eq. (8), we have *n*_*i*_=*i*+*n*_*d*_ and *n*_*m*_=*l*−(*i*+2*n*_*d*_). For a specific *n*_*d*_, the probability of a random walk producing diagonal shift *i* can be calculated as the number of the possible paths $ \binom {l}{i+2n_{d}} \cdot \binom {i+2n_{d}}{i+n_{d}} $, times the probability product of all insertions, deletions, and no shift change at each step $ q^{n_{d}}q^{n_{d}+i}(1-2q)^{l-\left (i+2n_{d}\right)} $. To calculate the probability of generating a diagonal shift *i* given *l*, *P*[*i*,*l*], we need to consider all possible values of *n*_*d*_, which is from 0 (no deletion) to (*l*−*i*)/2 (no match). So we have:

9 $$ {\begin{aligned}  {\mathcal{P}[i, l] \,=\, {\sum\nolimits}_{n_{d} = 0}^{(l - i)/2}{l \choose i+2n_{d}} \!\cdot\! {i+2n_{d} \choose i+n_{d}} \cdot q^{n_{d}}q^{n_{d}+i}(1\,-\,2q)^{l-\left(i+2n_{d}\right)}} \end{aligned}}  $$

To calculate *δ*, we sum up the probabilities $\mathcal {P}[i, l]$ for *i*=0,±1,±2,...,±*l* until we reach the level 1−*α*. We refer the reader to the original article [25] for a more detailed discussion of the model and a practical implementation using generating functions.

In our experiment, we set the sequencing accuracy *p*=0.85 and the indel rate *q*=0.06, as PacBio reads tend to have higher indel rates than substitution error rates. Users can adjust these parameters based on their data properties. The significance level *α* is 0.05 and *k* is 9. Using Eq. 6, we can obtain *ρ*=54. Using Eq. 9 and *ρ* as an estimation of *l*, we can obtain *δ*=5 given *ρ*=54. Thus, when *k*=9, seeds with inter-seed distance *D*((*i*_1_,*j*_1_),(*i*_2_,*j*_2_))≤54 and diagonal shift |*d*(*i*_1_,*j*_1_)−*d*(*i*_2_,*j*_2_)|≤5 are clustered in the same group. In addition, if *k*-mers (*i*_1_,*j*_1_) and (*i*_2_,*j*_2_) are in the same group and *k*-mers (*i*_2_,*j*_2_) and (*i*_3_,*j*_3_) are in the same group, we will cluster (*i*_1_,*j*_1_) and (*i*_3_,*j*_3_) as well.

### Group chaining

With group hit criteria, GroupK can find short similar regions. To identify the overlapping region, we aim to find a chain of group seed matches that maximizes the number of matched bases. We used the modified sparse dynamic programming for chaining [31, 32].

After generating a chain of group seed matches, we need to determine whether this chaining defines an overlap. We develop two criteria for this purpose. First, we calculate the expected number of matched bases from the group hit criteria, assuming that the chain covers the possible overlapping region with length *L*_*O*_ (*L*_*O*_ can be estimated by the extension of both ends of the optimal chain). We used the following equation to calculate the expected number of the matched bases *n*_*e*_:

10 $$ n_{e} = \frac{1}{c}\cdot\frac{L_{O}}{\rho}\cdot k  $$

Where *c* is a coefficient to control the criteria, *k* is the size of *k*-mer, *ρ* is the group hit criteria for the inter-*k*-mer distance. We only report the chaining result if the number of matched bases *n*≥*n*_*e*_. Second, we require that both reads have similar sizes inside the overlapping region.

**Fig. 6:**
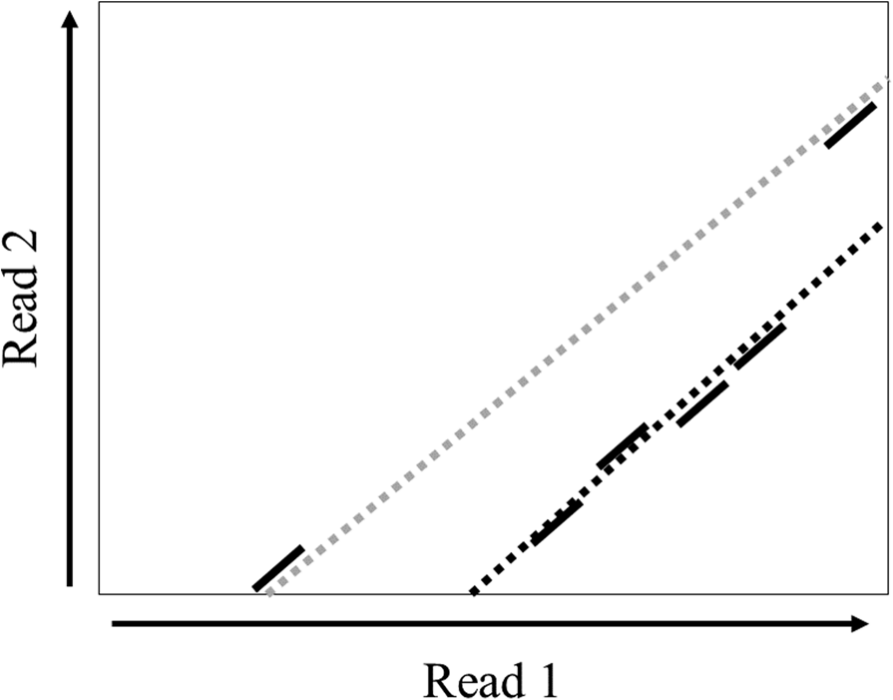
An example of the overlap size estimation. The suffix of read 1 and the prefix of read 2 form an overlap. Each short solid line represents a group seed match in the optimal chain. The black dashed line indicates the true overlap alignment region between the two reads. The gray dashed line, which is formed by the two ending group seeds in the optimal chain, can overestimate the overlap size

In our experiment, we found that sometimes using the optimal chain generated from sparse dynamic programming may overestimate the overlap region, as shown in Fig. 6. This overestimation can jeopardize the sensitivity of detecting small overlaps. We fix this problem by only keeping the collinear group seeds, which are used to estimate the overlap size.

### Implementation details of the major components

**Filtration by *k*-mer counts** In the first step of our pipeline, we use *k*-mer counting-based filtration to remove large numbers of read pairs that are not likely sequenced from the same loci on the underlying genome. We implemented *k*-mer counting using a generalized suffix array and the derived longest common prefix (LCP) array. The generalized suffix array *SA* is created from the concatenated reads (delimited by special characters such as $) using a linear algorithm [33]. Then, we create the LCP using both the suffix array *SA* and the reversed suffix array *SA*^′^ [34, 35]. Let the sequence of concatenated reads be *T*. Following the definition of the reversed suffix array, for a suffix starting at position *SA*[*i*], we have *SA*^′^[*SA*[*i*]]=*i*. For each position *i* in the LCP, LCP[i] contains the size of the longest common prefix between *SA*[*i*] and *SA*[*i*−1]. The key observation [33] for efficient computation of LCP[i] is: for a position *j* in *T*, if *LCP*[*SA*^′^[*j*−1]] is *L*, *LCP*[*SA*^′^[*j*]]≥*L*−1. The whole LCP array construction takes linear time to the size of *T* [33].

In order to count the shared *k*-mers between reads and also report read pairs passing the *k*-mer counting threshold, we use both LCP and an auxiliary data structure recording the read IDs (denoted as array *readID*). For a suffix starting at position *SA*[*i*], its read ID is at *readID*[*i*]. The pseudocode of finding the number of shared *k*-mers can be found in Algorithm 1. In practice, we also count *k*-mers between a read and the other read’s reverse complement.


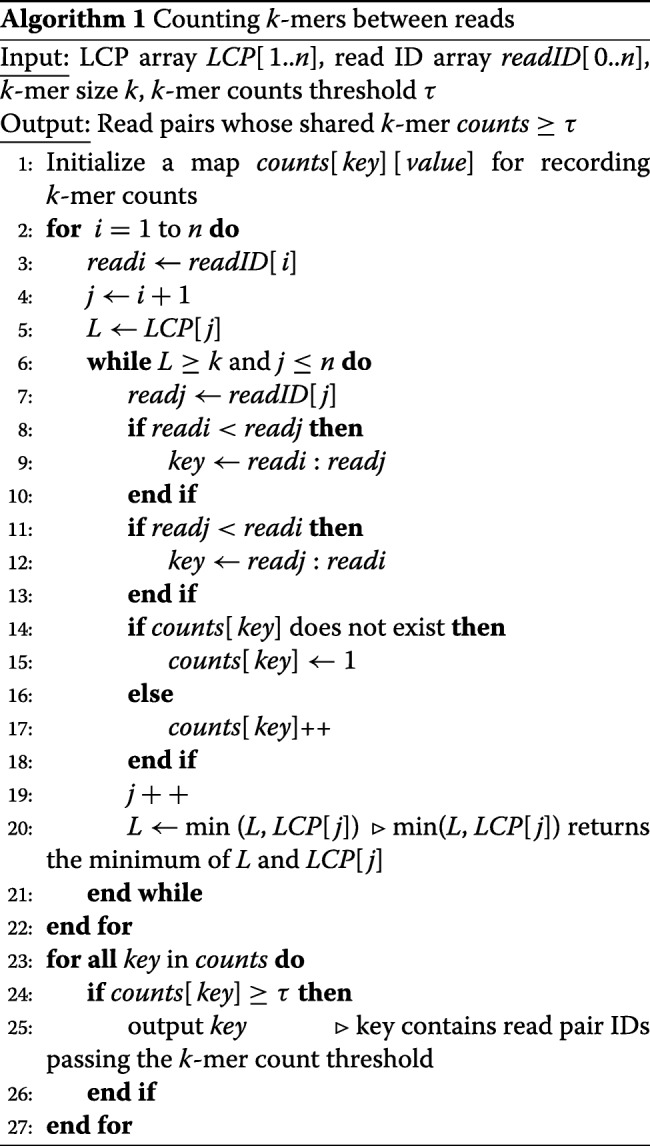


**Group seed match and chaining** Any pair of reads that pass the above filtration stage will be used as input for finding group seed matches. All other pairs will be discarded. Currently, we are using the codes of YASS [36, 37] for finding the group seed matches. The program uses hashing table to find short exact matches and creates the groups of matches on the fly. It is our future work to re-implement group seed matching using more efficient indexing-based methods. Implementation of chaining algorithm has been modified from the program of global chaining algorithm in SeqAn library [38].

**Time complexity analysis** If we have *N* reads with average read length *L*, our text size is *NL*. Therefore, we have *NL* elements in the suffix array and the corresponding LCP array. For each suffix in the suffix array, suppose on average, it can form LCPs with *m* other suffixes with size above *k*, which is the size of *k*-mer used in the *k*-mer-count filtration steps. So the time complexity of finding all the shared *k*-mers for all possible reading pairs is $ \mathcal {O}(mNL) $. If *k* is large enough (e.g., *k*=15 and 11 in our experiment), we have *m*≪*NL*, so the time complexity will be dominated by *NL*.

For *N*^′^ read pairs that pass the filtration stage, let the average number of *k*-mer hits for each pair be *q*. Sorting the hits will need $ \mathcal {O}(q\log q) $ and iterating through all hits to find groups is linear to *q*. For all the read pairs, the time complexity is $ \mathcal {O}(N'q\log q) $.

Assuming finally we have *r* group seeds, the chaining procedure has complexity in $ \mathcal {O}(r\log r) $ for each read pairs. For all the read pairs, the time complexity is $\mathcal {O}(N'r\log r) $. It is practically very fast because the number of group seed matches is very small compared to the original seed hits (indicated by Fig. 3).

## Results

We focus on evaluating the sensitivity and precision of overlap detection. We applied GroupK to three PacBio datasets and one ONT dataset: a simulated PacBio RSII *E. coli* sequencing dataset, a real PacBio RSII *E. coli* sequencing dataset, a PacBio RSII human foot metagenomic sequencing dataset, and an ONT *E. coli* (SQK-MAP-006) dataset. For simulated *E. coli* dataset, we have the true sampling position for each read as our ground truth. For the real *E. coli* dataset and human foot dataset, we determine the ground truth via BLASR’s [18] alignments against the reference genome.

We benchmarked GroupK’s performance with Minimap [16], Minimap2 [14], DALIGNER [17], MHAP [15], and GraphMap [19]. Those tools and methods are representative overlap detection tools for long erroneous reads from PacBio or ONT [13]. All the detailed parameters can be found at the website listed in “Availability of data and materials”. Our main metrics include: (1) sensitivity, which measures the ratio of the true overlaps identified by each program to the whole set of overlapping pairs; (2) precision, which quantifies the ratio of true overlap detected by each program to the total reported overlapping pairs; and (3) F1 score, which is the harmonic mean of sensitivity and precision. A reported overlapping pair is regarded as correct if it is also present in our ground truth. The detailed overlap region and overlap length were not considered in the current evaluation because these read pairs can go through a more accurate alignment program for generating the final overlap alignment. As we discussed in Background, our goal is to identify overlapping reads without using error correction. All tested tools will thus be applied to their raw data set.

### Simulated *E. coli* dataset

We first evaluated the performance of our method on a simulated *E. coli* dataset. The dataset was generated using PBSIM [26] with *E. coli* K-12 MG1655 as the reference genome [39]. The length distribution and the quality profile were derived from real PacBio P6-C4 *E. coli* dataset [40]. The simulated dataset has 5620 reads, with average length of 8,344.78 bps and 14.5% average error rate (8.6% insertions, 4.4% deletions, and 1.4% substitutions).

From the report by PBSIM, we can obtain the exact locations where the simulated reads are sampled in the genome. This information provides us with the ground truth for reads’ overlaps so that we can calculate the sensitivity and precision.

Following the pipeline we discussed in Methods, we first used *k*-mer-counting as the filtration stage. According to Eq. 1, Eq. 3, and Fig. 5, we discarded all read pairs with less than two 15-mer matches. The sensitivity of the filtration is 0.979 and only about 6% of read pairs are kept for downstream analysis.

**Fig. 7:**
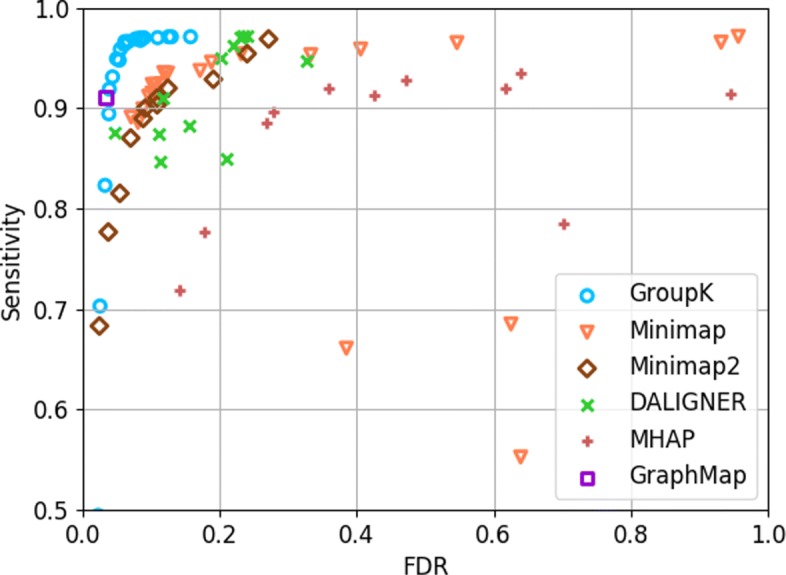
The ROC-like plot using GroupK, Minimap, Minimap2, DALIGNER, MHAP, and GraphMap on the simulated PacBio *E. coli* dataset. The x-axis represents the false discovery rate (FDR=1−precision). Y-axis is the sensitivity (0.5 to 1)

We evaluated the performance of our tool by adjusting the group seed match criteria coefficient *c*, which is introduced in Methods. With the increase of *c*, sensitivity will become higher, and the precision will become lower. As shown in Fig. 7, GroupK can achieve 5 to 6% improvement on the sensitivity with similar precision to other overlap detection tools.

#### Running time and memory usage

We evaluated the running time and the peak memory usage of the tested tools in this experiment. We run all overlap detection tools with a single core of 2.4Ghz 14-core Intel Xeon E5-2680v4 CPU and 32 GB memory requested from the High-Performance Computing Center at Michigan State University. The performance is measured with the best F1 score. The results are reported in Table 1. For the memory usage, Minimap2 is the most efficient one but all others are comparable. GroupK is slower than other tools, partially because we use small *k*-mers. We found that the bottleneck of our program is the group matching stage, which accounts for about 1200 of 1871 s. By implementing a more efficient indexing-based method, we expect to reduce the running time of this stage. For example, we can speed up *k*-mer counting by adopting the method used in KMC [41].

**Table 1:** Computational performance on the simulated *E. coli* dataset

	GroupK	Minimap	Minimap2	DALIGNER	GraphMap	MHAP
Time (seconds)	1871	30	16	39	171	858
Memory (GB)	1.994	1.754	1.097	2.288	1.873	2.562

The computational performance of overlap detection using GroupK, Minimap, Minimap2, DALIGNER, MHAP, and GraphMap on the simulated *E. coli* dataset

### Real PacBio *E. coli* dataset

After using the simulated dataset to evaluate our method’s performance, we applied GroupK to a real PacBio RS II (P6-C4) *E. coli* dataset [40]. The coverage of the whole dataset is 150X. To test the performance of low coverage data, we sampled a 15X coverage dataset based on the read length distribution of the whole dataset. The dataset has 14,262 reads, with the average length of 4,882.09 bps and average error rate of 14.14% (error rate is estimated using quality score).

We applied BLASR to map the reads to the reference genome to estimate the ground truth (BLASR was run with parameters: minReadLength, 2000; maxScore, 1000; maxLCPLength, 16; minMatch, 12; m 4 and nCandidates/bestn set to 10 × sequencing coverage). The mapping result from BLASR may contain short alignments due to the repeat in the genomes. So when we determine the ground truth, we only consider the alignments that cover at least 80% of the read. By removing short noisy alignments, we can make sure the BLASR alignments are close to the underlying ground truth.

We used the same filtration setup adopted in the simulated *E. coli* experiment. Among all overlap detection tools we tested, GroupK still achieved the highest sensitivity with comparable precision (Table 2). With slightly higher precision, our sensitivity is 4% better than the next best tool, Minimap. Compared to the previous experiment, the difference in sensitivity is smaller. One reason lies in the construction of the ground truth dataset. In the simulated dataset, we used the sample positions of all reads to determine whether two reads form an overlap. Thus, that dataset can include reads with small overlaps or reads with higher error rates. In this dataset, our method discarded BLASR alignments with high error rates and the remaining alignments have higher similarities with the reference genome and thus produce fewer “hard cases”. As Minimap is the second best tool for this dataset, we further analyzed the performance of GroupK and Minimap on read pairs of different overlap size in the next section.

**Table 2:** Overlap detection on the real *E. coli* dataset

	GroupK	Minimap	Minimap2	DALIGNER	GraphMap	MHAP
Best F1 score	**0.9311**	0.9037	0.8426	0.8340	0.7758	0.7023
Sensitivity	**0.9330**	0.8939	0.8778	0.9066	0.6741	0.7258
Precision	**0.9292**	**0.9138**	0.8101	0.7722	**0.9137**	0.6802

The performance of overlap detection using GroupK, Minimap, Minimap2, DALIGNER, MHAP, and GraphMap on the real PacBio RS II (P6-C4) *E. coli* dataset. Here we only report the experiment results with the highest F1 score for each tool

**Fig. 8:**
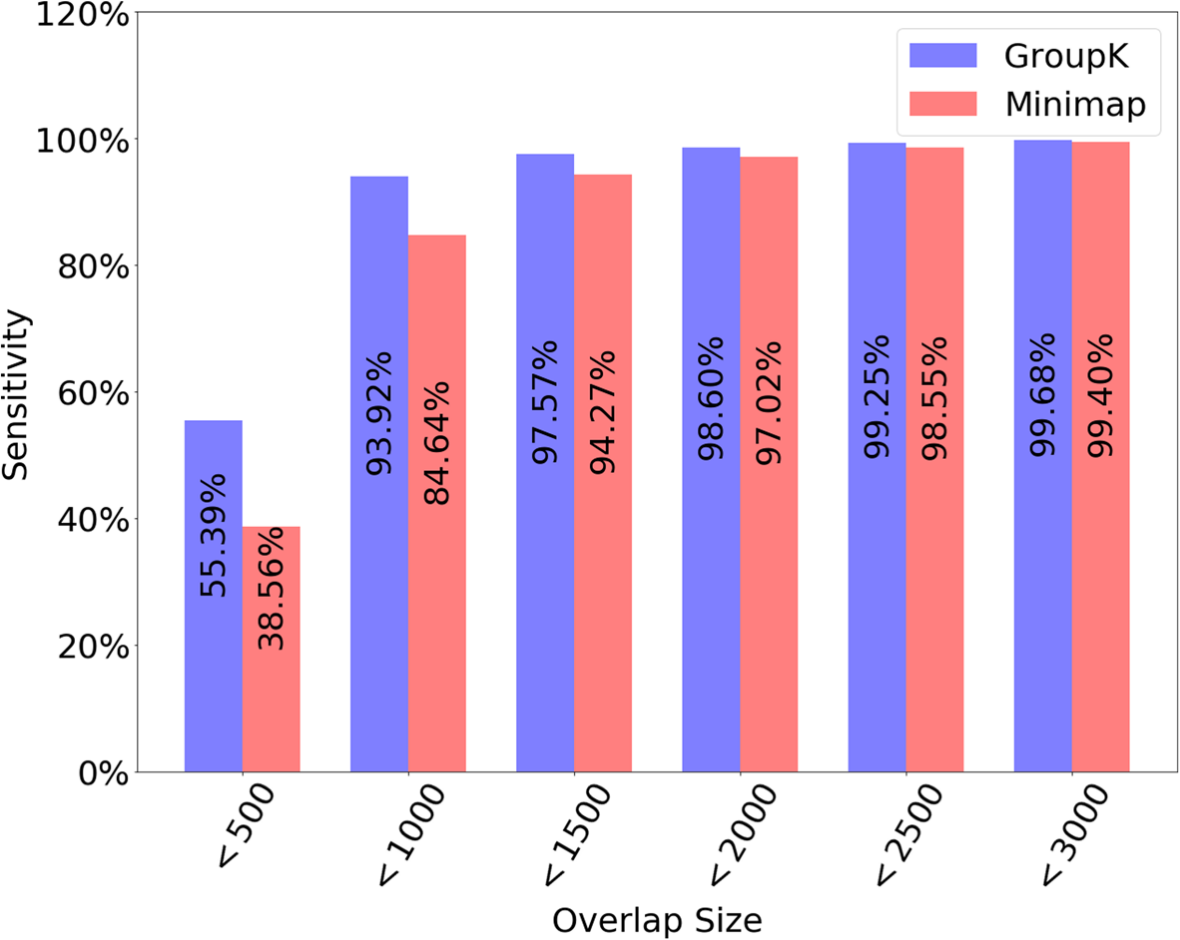
Sensitivity of GroupK and Minimap for detecting overlaps of different size on PacBio *E. coli* dataset. The x-axis represents the overlap size. Y-axis is the corresponding sensitivity of the bin. The X-axis bin width is 500 and the figure only included the first 6 bins (i.e. up to overlap size 3000) as their sensitivity becomes more similar with the increase of the overlap size

**Performance with different overlap size** We divide all overlapping pairs into bins of width 500 based on the overlap size. For example, the first bin has the read pairs with overlap sizes from 0 to 499, and the second bin has read pairs with overlap sizes from 500 to 999, and so on. For each bin, we compared the sensitivity of GroupK and Minimap using parameters yielding the similar precision in Fig. 8. For Minimap, we showed the result with the highest F1 score. For GroupK, we selected a parameter so that it achieves similar precision to Minimap (F1 score: 0.9241, sensitivity: 0.9344, precision: 0.9140). According to Fig. 8, GroupK has much better sensitivity when the overlap size is less than 2000. As we showed in Fig. 1, there are a significant number of overlaps with overlap size smaller than 2000 even for 30X coverage. Being able to identify small overlaps allows us to generate more complete assemblies using long reads. This is particularly useful for low coverage data, such as what we usually have in metagenomic datasets.

### Human foot metagenomic dataset

One of the major utilities of our tool is to identify overlaps between reads in metagenomic data that are sequenced using the PacBio platform. For complicated microbial communities, metagenomic data containing only short reads poses serious computational challenges for assembly and the downstream composition/functional analysis. Long reads hold the promise to produce more complete and accurate microbial genome assemblies for the metagenomic dataset. In this experiment, we evaluated the performance of overlap detection for a mock metagenomic dataset constructed from a real human foot dataset [5]. A particular challenge for this experiment is the low coverage of the component species in the metagenomic dataset, which could be caused by sequencing throughput and complexity of the sample.

The human foot sample was sequenced by linear PacBio RSII TdT (terminal deoxynucleotidyl transferase). Sequences that can be mapped to the human genome were removed as host-derived DNA. According to the Supplementary Materials of [5], there are about 1,000 bacteria and viruses in this metagenomic dataset. However, we cannot evaluate the performance of overlap detection on all the reads from the 1000 microbes because the coverages of many species are too low to yield meaningful overlaps. In order to construct the ground truth, we need to align the reads against species with known reference genomes and reasonable coverage. Thus, we only choose reads satisfying the following criteria: 1) the reads are sequenced from a species with known reference genome; and 2) the coverage of the species cannot be too small (e.g., >3X coverage). Based on these criteria, we keep the reads sequenced from three bacteria: *Corynebacterium aurimucosum* (6.3X Coverage), *Corynebacterium tuberculostearicum* (8.5X Coverage), and *Staphylococcus hominis* (3.2X Coverage). The reads are recruited via BLASR. The alignment positions are used to determine which reads form an overlap. Note that *Corynebacterium aurimucosum* and *Corynebacterium tuberculostearicum* belong to the same genus and may contribute to the false positive overlap detection due to their shared regions. The average length of the reads is 1696.25 bps, which is much shorter than the reads in the previous experiments.

As this dataset contains much shorter reads, the expected number of *k*-mer hits will change. Intuitively we need to use shorter *k*-mers to ensure high filtration sensitivity. Using the read length distribution, we calculated *E*[*X*_*r*_] (Eq. 1) and *E*[*X*_*o*_] (Eq. 3) and determined the *k*-mer counting-based filtration criteria. In this experiment, we only kept read pairs that share at least three 11-mers.

For this mock metagenomic dataset, GroupK yielded significantly better performance than other tools on metrics including F1 score, sensitivity, and precision (Table 3). Compared to other tools, GroupK can produce much higher sensitivity without sacrificing precision, leading to the higher F1 score. Besides evaluating the performance of various tools on all the reads from the three species, we also reported the performance of different overlap detection tools on each single bacteria dataset without mixing with other species (Table 3). In these tools, GraphMap has high specificity for all three with sacrifice of sensitivity. However, GroupK still achieves the best performance overall. The comparisons suggest that our method has great potential to detect overlaps for data with very low coverage (around 5X). This will enable better assembly for PacBio sequenced metagenomic data, which will become more available with the advances of long read sequencing technologies.

**Table 3:** Overlap detection on the human metagenomic dataset

	GroupK	Minimap	Minimap2	DALIGNER	GraphMap	MHAP
Total:						
Best F1 score	**0.9163**	0.8306	0.8776	0.6911	0.7188	0.7812
Sensitivity	**0.8954**	0.7802	0.8352	0.6012	0.5721	0.6803
Precision	0.9381	0.8880	0.9245	0.8027	**0.9666**	0.9174
*C. aurimucosum*:						
Best F1 score	**0.9512**	0.8072	0.9117	0.7432	0.7397	0.8545
Sensitivity	**0.9228**	0.6858	0.8467	0.6266	0.5892	0.8045
Precision	**0.9814**	**0.9806**	**0.9874**	0.9131	**0.9937**	0.9111
*C. tuberculostearicum*:						
Best F1 score	**0.9454**	0.8688	0.9050	0.8315	0.7276	0.7961
Sensitivity	**0.9105**	0.7958	0.8346	0.7150	0.5727	0.7355
Precision	**0.9830**	0.9567	**0.9884**	**0.9934**	**0.9977**	0.8675
*S. hominis*:						
Best F1 score	**0.9163**	0.7651	0.8024	0.8754	0.6343	0.6861
Sensitivity	**0.8733**	0.6887	0.6813	**0.7967**	0.4658	0.6348
Precision	0.9245	0.8606	0.9759	0.9713	**0.9938**	0.7464

The performance of overlap detection using GroupK, Minimap, Minimap2, DALIGNER, MHAP, and GraphMap on the mock metagenomic dataset. Here we only report the experimental results with the highest F1 score for each tool

### Real ONT *E. coli* dataset

We also tested our method on one ONT dataset. We used downsampled 15X coverage 2D reads from the SQK-MAP-006 dataset as 2D reads provide higher quality than 1D reads. We followed the same pipelines we used for the real *E. coli* PacBio dataset. As 2D ONT reads have similar error rates to the PacBio reads, we expect that our tool can still achieve reasonable performance given the same setup for the PacBio dataset. Therefore, we used the same parameters as the ones we used for the PacBio *E. coli* dataset.

Among these tools, GraphMap was designed for ONT data, Minimap2 provides a specific setup for finding the overlap on ONT dataset. All other tools are not specifically designed for ONT datasets. GroupK achieves the best F1 score compared to other tools’ default setup while keeping the highest sensitivity (Table 4). This result suggests that our strategy is robust with different types of long reads.

**Table 4:** Overlap detection on the ONT *E. coli* dataset

	GroupK	Minimap	Minimap2	DALIGNER	GraphMap	MHAP
F1 score	**0.9383**	**0.9310**	0.9090	0.8272	**0.9280**	0.8122
Sensitivity	**0.9597**	**0.9546**	0.9362	0.8730	0.8991	0.8871
Precision	0.9178	**0.9085**	0.8833	0.7860	**0.9589**	0.7490

The performance of overlap detection using GroupK, Minimap, Minimap2, DALIGNER, MHAP, and GraphMap on the real ONT SQK-MAP-006 *E. coli* dataset. Minimap2 uses the ava-ont setup, which is optimized for ONT data

## Discussion

Seeding is a key step for overlap detection because of the high error rate of long reads. Successful seeding strategies should balance the sensitivity and the specificity to achieve the optimal performance. Popular seeding methods include maximal exact matches, spaced seeds, and gapped spaced seeds. However, to successfully find a hit between two reads, these methods still need either to find relatively long continuous exact matches (large *k*-mer) or to find inexact matches following certain error patterns (spaced seed). Compared to these methods, group seed matching is more flexible as it requires multiple short exact matches without specifying the error patterns. This flexibility leads to high sensitivity, and meanwhile the specificity is still guaranteed with the group seed match criteria.

Currently the group seed matching step based on hash table is the bottleneck of our overlap detection pipeline. A new method that can improve the running time efficiency of this step is needed to make the algorithm achieve the same speed as other faster overlap detection tools.

## Conclusions

In this work, we developed an overlap detection tool for third-generation sequencing data. By adopting the group hit criteria to cluster a group of short *k*-mer hits that satisfy statistically derived distance constrains, our method can improve the sensitivity of overlap detection without sacrificing precision. Our experimental results have shown that for datasets with low sequencing coverage, our program can detect significantly more overlapping pairs while keeping high precision. One utility of our approach is to detect small overlaps between long reads of rare species in a microbial community.

## Abbreviations

BWT: Burrows–wheeler transform
LCP: Longest common prefix
ONT: Oxford Nanopore
PacBio: Pacific biosciences
SMRT: Single molecule real time sequencing
TdT: Terminal deoxynucleotidyl transferase
